# Combined preoperative prognostic nutritional index and D-dimer score predicts outcome in colorectal cancer

**DOI:** 10.1186/s12893-023-01925-8

**Published:** 2023-02-07

**Authors:** Shibin Zhu, Jianyuan Yin, Qianwen Ye, Jia Xiang, Zihao Zhang, Bing Yan

**Affiliations:** 1 Department of Clinical Laboratory, Hainan Hospital of Chinese PLA General Hospital, Sanya, Hainan 572000 People’s Republic of China; 2 Department of Critical Care Medicine, Hainan Hospital of Chinese PLA General Hospital, Sanya, Hainan 572000 People’s Republic of China; 3 Department of Oncology, Hainan Hospital of Chinese PLA General Hospital, No. 80 of Jianglin Road, Haitang District, Sanya, Hainan 572000 People’s Republic of China

**Keywords:** Colorectal cancer, Prognostic nutritional index, D-dimer, Score, Prognosis

## Abstract

**Background:**

The prognostic nutritional index (PNI) and D-dimer (DD) levels represent useful prognostic indicators in colorectal cancer (CRC); however, a combination of these indicators, namely, the PNI and DD score (PDS) was less addressed.

**Methods:**

A retrospective study with 183 patients after curative surgery was conducted. Patients were divided into 3 subgroups: PDS 0, decreased PNI and increased DD levels; PDS 1, decreased or increased PNI and DD levels; PDS 2, increased PNI and decreased DD levels. The differences in disease-free survival (DFS) and overall survival (OS) were compared among these subgroups, and risk factors for outcome were determined.

**Results:**

A total of 56, 65 and 62 patients were assigned to the PDS 0, 1 and 2 subgroups, respectively. PDS was significant in predicting both the DFS (area under the curve (AUC) = 0.68, P < 0.001) and OS (AUC = 0.74, P < 0.001). PDS 0 patients were more likely to be associated with old age (P = 0.032), laparotomy (P < 0.001), elevated CEA (P = 0.001), T_3_ + T_4_ (P = 0.001) and advanced TNM stage (P = 0.031). PDS 0 patients had significantly inferior DFS (log rank = 18.35, P < 0.001) and OS (log rank = 28.34, P < 0.001) than PDS 1 or 2 patients. PDS was identified as an independent risk factor for both DFS (PDS 1: HR = 0.54, 95% CI: 0.30–1.00, P = 0.049; PDS 2: HR = 0.40, 95% CI: 0.20–0.79, P = 0.009) and OS (PDS 1: HR = 0.44, 95% CI: 0.22–0.88, P = 0.020; PDS 2: HR = 0.17, 95% CI: 0.06–0.45, P < 0.001).

**Conclusion:**

The PDS is a useful prognostic indicator for CRC patients after curative surgery, and PDS 0 patients have inferior survival. Additional future studies are needed to validate these findings.

## Background

Colorectal cancer (CRC) is the third most lethal cancer in the world with a heavy health burden in China, where an estimated 592,232 new cases were diagnosed and up to 309,114 deaths occurred in 2022 [1]. Globally, the incidence of CRC has more than doubled with a corresponding remarkable increase in deaths noted between 1990 and 2019 [2]. Although an increasing number of patients are diagnosed and cured at a very early stage due to sigmoidoscopy screening [3], the 5-year survival rate for those with stage II and III disease remains unsatisfactory [4].

It is well established that the outcome of cancer patients is determined by many factors, including nutrition status [5] and anticancer immunity [6], in addition to the cancer cells themselves; accordingly, it is plausible that prognostic markers could be more reasonable if these factors are comprehensively considered. The prognostic nutritional index (PNI), which is a marker calculated based on serum albumin and lymphocytes, is a robust prognostic indicator in many malignancies, including lung cancer [7], nasopharyngeal cancer [8], liver cancer [9], breast cancer [10], gastric cancer [11] and CRC [12, 13]. Interestingly, previous studies have indicated that the prognostic efficacy of PNI was superior to that of other inflammation-based prognostic indicators [14, 15]. In CRC, the PNI was also noted to be a better marker than other inflammatory indicators and was the only independent risk factor for survival in stage IIA or III cases [16, 17]. Nonetheless, it was notable that PNI alone was limited by its prognostic efficacy. Specifically, the area under the curve (AUC) ranged from 0.56 to 0.67 in non-metastatic cases with a relatively low sensitivity (58.6%) and specificity (59.6–78.3%) [17, 18] and was only 0.62 in metastatic cases [19]. In recent years, some studies have reported a combination of PNI with other markers and found moderately improved AUCs in predicting the outcome. For example, the PNI was combined with the albumin-to-globulin ratio as a prognostic indicator of esophagogastric junction cancer [20] and with hemoglobin to predict the prognosis of esophageal squamous cancer [21]. In recent years, the pivotal role of circulating tumor cells (CTCs) in patients who relapse after surgery has become increasingly popular, and these cells are considered to be a strong prognostic indicator in stage I–III cases of CRC [22, 23]. Interestingly, a study reported that a combination of the controlling nutritional status score (an index calculated based on serum albumin, lymphocyte counts and total cholesterol that is similar to PNI) and CTCs potentially exhibits superior prognostic efficacy and is able to distinguish the survival of subgroups [24]. However, of note, the detection of CTCs was limited by specific technology and was not routinely conducted in practice; other alternative indicators are still needed.

D-dimer (DD), which is an end product in the biological process of fibrin degradation, is a sensitive indicator of coagulation and fibrinolysis. Interestingly, DD also has important value in many cancers [25–27]. Similar to CRC, DD has long been established as a useful tumor marker, and its diagnostic value in preoperative staging is comparable to that of carcinoembryonic antigen (CEA) [28]. The prognostic value of DD in CRC has also been well studied [29–31] and was demonstrated to be even better than CEA in metastatic scenarios [32]. More importantly, some previous studies indicated that the level of DD was closely correlated with CTCs in some cancers [33, 34]. Based on these facts, we hypothesize that a combination of PNI and DD could have good prognostic efficacy in CRC; however, related studies are limited.

Here, we aimed to explore the prognostic role of a combination of PNI and DD score (PDS) in CRC patients after curative surgery.

## Methods

### Patients

Consecutive patients who underwent curable surgery for colorectal adenocarcinoma at Hainan hospital of Chinese PLA general hospital from December 2012 to May 2020 were enrolled retrospectively. Cases meeting any one of the following criteria were excluded: 1. any preoperative anticancer therapies; 2. suspected distant lesions identified by imaging examinations; 3. complications that require long-term administration of anticoagulant drugs, such as aspirin and Plavix; 4. lack of preoperative laboratory tests, such as DD; 5. absence of postoperative pathological TNM message; and 6. follow-up problems. Parameters including age, sex, tumor location and others were also recorded as previously described [35, 36]. The study was approved by the ethics committee of Hainan Hospital of Chinese PLA General Hospital (ID: 301HLFYLS15), and informed consent was obtained from the patients or their authorized relatives.

### Calculation of PDS and other systematic inflammatory prognostic indicators

Routine blood tests were conducted in the departmental clinical laboratory as previously described [35]. The reference was 35–50 g/L for serum albumin, 1.75–7.00 × 10^9^/L for peripheral absolute lymphocyte counts, and 0–500 ng/mL for DD levels. All tests were performed within one week before curative surgery as previously described [35, 36]. The PNI was calculated as previously described [37], and patients were divided into PNI-low or -high and DD-low or -high subgroups based on the optimal cutoff points described in the statistical results below. Subsequently, patients were divided into 3 subgroups: PDS 0, PNI low and DD high; PDS 1, PNI/DD low or PNI/DD high; and PDS 2, PNI high and DD low. Additional systematic inflammatory prognostic indicators, such as the neutrophil to lymphocyte ratio (NLR), lymphocyte to monocyte ratio (LMR) and platelet to lymphocyte ratio (PLR), were also calculated according to a previous report [37].

### Follow-up procedures and definition of disease-free survival (DFS) and OS

Patients were routinely followed according to our previous studies [36]. DFS was determined based on the date of surgery to the date of any relapse or distant metastasis or the date of death from any cause, and OS was determined from the same point to the point of any cause of death. The final follow-up point was December 2021.

### Data analysis

The data were processed using SPSS 20.0 (SPSS Inc., Chicago, IL, USA), MedCalc v19.0.7 (MedCalc Software Ltd., Ostend, Belgium) and GraphPad Prism 5 (GraphPad Software Inc., San Diego, CA, USA). The optimal cutoff points of PNI and DD for the outcome were determined by receiver operating characteristic curve (ROC) analysis. Differences in other systematic inflammatory prognostic indicators in PDS subgroups were analyzed by one-way ANOVA followed by Bonferroni test for subgroup comparison. Survival differences among PDS subgroups were analyzed by Kaplan–Meier analysis and log-rank tests. Risk factors for survival were determined using a Cox proportional hazards model with the iterative forward LR method. All tests were two-sided with P < 0.050 considered statistically significant.

## Results

### Demographic characteristics of the study cohort and the prognostic efficacy of PDS

**Fig. 1:**
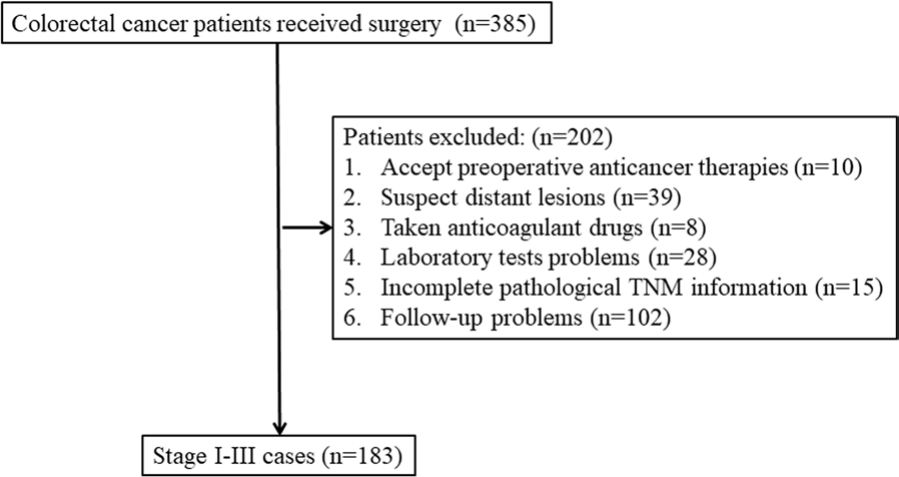
Patient collection flowchart

**Fig. 2:**
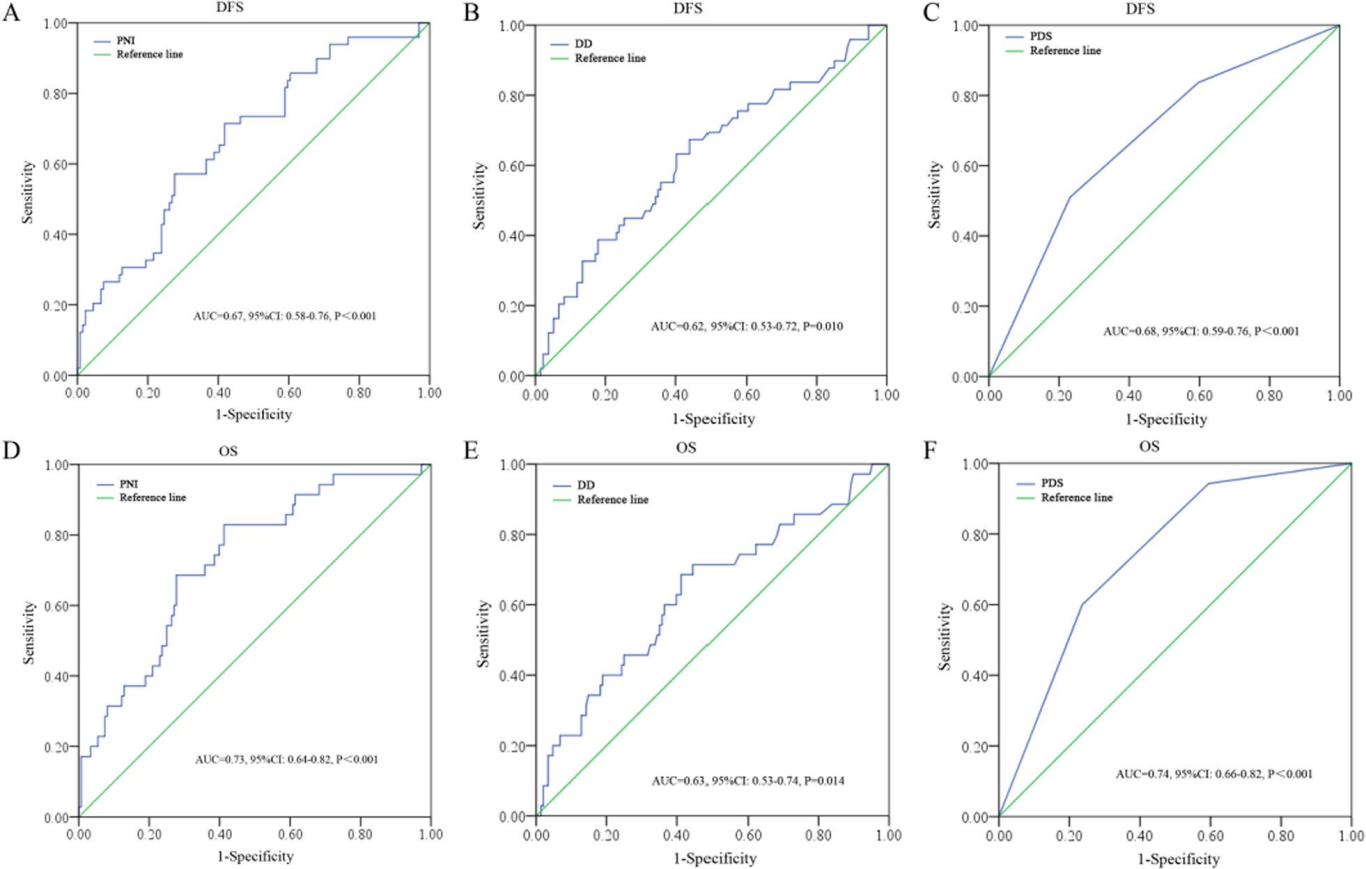
ROC analysis of PNI, DD and PDS in predicting DFS or OS

According to the exclusion criteria, a total of 183 cases were included in the study (Fig. 1). The study included 71 females and 112 males with a medium follow-up of 61.04 months (m). At the end of the 3-year period, 0, 12 and 23 patients died in stages I (n = 39), II (n = 77) and III (n = 67), respectively. The overall 3-year DFS rate and OS rate were 73.22% and 80.87%, respectively. Based on ROC analysis, the PDS was significant in predicting both the 3-year DFS (AUC = 0.68, 95% CI: 0.59–0.76, P < 0.001) and OS (AUC = 0.74, 95% CI: 0.66–0.82, P < 0.001) (Fig. 2).

### Differences in clinicopathological features in the PDS subgroups

Based on ROC analysis and the *Youden index* (3-year OS served as the end-point), patients were divided into PNI high (≥ 48.21 g/L) or low (< 48.21 g/L) subgroups and DD high (≥ 139.50 ng/mL) or low (< 139.50 ng/mL) subgroups. Based on the aforementioned algorithm, 56, 65 and 62 patients were assigned to the PDS 0, 1 and 2 subgroups, respectively. PDS 0 patients were more likely to present with old age (P = 0.032), laparotomy (P < 0.001), elevated CEA (P = 0.001), T_3_ + T_4_ (P = 0.001) and advanced TNM stage (P = 0.031); no other clinicopathological features were significantly different among PDS subgroups (Table 1).

**Table 1:** Differences for the clinicopathological features among PDS subgroups

Features	PDS subgroups				
	Patient No	PDS 0	PDS 1	PDS 2	P
Age (year)					0.032^*^
< 60	85	18	33	34	
≥ 60	98	38	32	28	
Sex					0.181
Male	71	18	31	22	
Female	112	38	34	40	
Type of resection					< 0.001^*^
Laparotomy	29	17	5	7	
Laparoscopy	154	39	60	55	
Tumor sites					0.128
Right	43	18	15	10	
Left	140	38	50	52	
Histological grade					0.103
Well + moderate	158	44	57	57	
Poor	25	12	8	5	
Tumor morphology					0.129
Ulcerated type	103	33	37	33	
Protruded type	43	9	15	19	
Mixed type	30	14	9	7	
Unknown	7	0	4	3	
Mucinous constituent					0.684
Without	152	48	52	52	
With	31	8	13	10	
Tumor deposits					0.090
Without	162	46	57	59	
With	21	10	8	3	
CEA level					0.001^*^
Normal	116	24	46	46	
Elevated	67	32	19	16	
Combined T stages					0.001^*^
T_1_ + T_2_	47	5	17	25	
T_3_ + T_4_	136	51	48	27	
Combined N stages					0.388
N_0_	112	31	39	42	
N_1+_N_2_	71	25	26	20	
TNM stages					0.031^*^
I	39	4	16	19	
II	77	29	25	23	
III	67	23	24	20	

^*^With significant statistical difference

### Correlation of PDS with other systematic inflammatory indicators

**Fig. 3:**
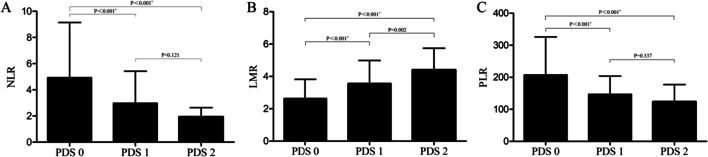
Differences in the NLR (**A**), LMR (**B**) and PLR (**C**) in the PDS subgroups

As presented in Fig. 3, significant differences for NLR, LMR and PLR were noted among different PDS subgroups. In general, PDS 0 patients had a significantly increased NLR and PLR and a decreased LMR compared with the other groups (all P < 0.001). For PDS 1 and PDS 2, only LMR (P = 0.002) was significantly different; NLR (P = 0.121) and PLR (P = 0.337) did not differ.

### Survival differences among PDS subgroups

**Fig. 4:**
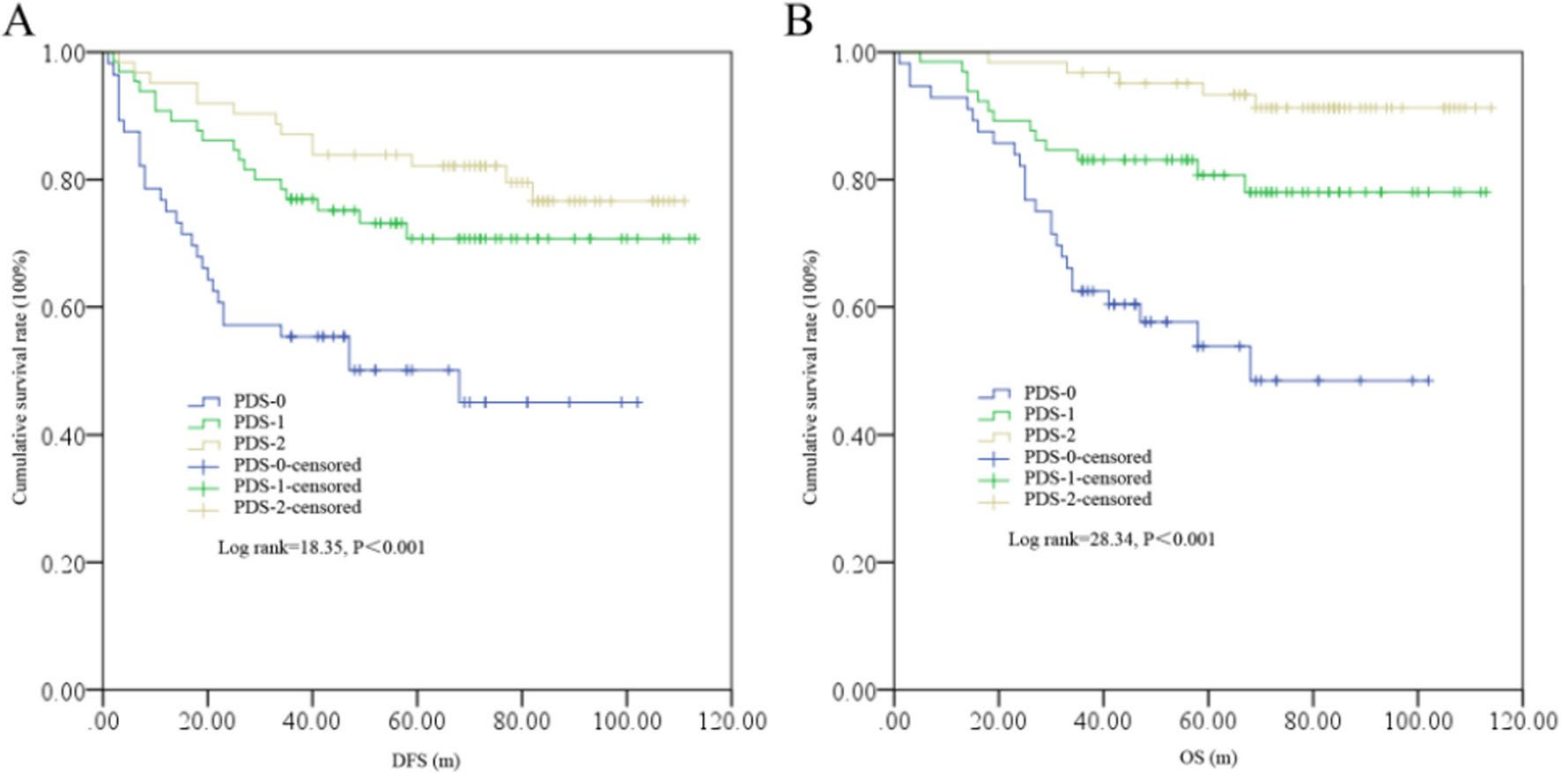
DFS (**A**) and OS (**B**) differences among the PDS subgroups

Based on Kaplan–Meier analysis, survival significantly differed among the PDS subgroups (Fig. 4). Specifically, PDS 0 patients had worse DFS and OS than PDS 1 and 2 patients; interestingly, OS but not DFS was also significantly different between the PDS 1 and 2 subgroups (log rank = 4.80, P = 0.029).

### Univariate and multivariate analyses of risk factors for survival

Using the Cox hazard model, the univariate test indicated that type of resection tumor morphology, tumor deposits, CEA level, combined T and N stages, TNM stages, and PDS were risk factors for both DFS and OS (Table 2). Further multivariate tests indicated that PDS was an independent risk factor for both DFS (PDS 1: HR = 0.54, 95% CI: 0.30–1.00, P = 0.049; PDS 2: HR = 0.40, 95% CI: 0.20–0.79, P = 0.009) and OS (PDS 1: HR = 0.44, 95% CI: 0.22–0.88, P = 0.020; PDS 2: HR = 0.17, 95% CI: 0.06–0.45, P < 0.001) (Table 3).

**Table 2:** Univariate tests for risk factors for DFS and OS

	DFS			OS		
	P	HR	95%CI	P	HR	95%CI
Age (years)						
< 60	1			1		
≥ 60	0.702	1.11	0.66–1.84	0.544	0.53	0.45–1.52
Sex						
Male	1			1		
Female	0.198	0.70	0.41–1.21	0.158	0.63	0.33–1.20
Type of resection						
Laparotomy	1			1		
Laparoscopy	0.005^*^	0.43	0.24–0.78	0.006^*^	0.39	0.20–0.76
Tumor sites						
Right	1			1		
Left	0.897	0.96	0.50–1.84	0.270	0.63	0.28–1.42
Histological grade						
Well + moderate	1			1		
Poor	0.060	1.88	0.97–3.62	0.052	2.08	0.99–4.34
Tumor morphology						
Ulcerated type	1			1		
Protruded type	0.044^*^	0.46	0.21–0.98	0.029^*^	0.31	0.11–0.89
Mixed + Unknown	0.932	0.97	0.52–1.83	0.807	1.09	0.54–2.19
Mucinous constituent						
Without	1			1		
With	0.290	1.41	0.75–2.66	0.276	1.51	0.72–3.14
Tumor deposits						
Without	1			1		
With	< 0.001^*^	5.98	3.35–10.70	< 0.001^*^	5.00	2.58–9.67
CEA level						
Normal	1			1		
Elevated	< 0.001^*^	2.90	1.73–4.86	< 0.001^*^	3.60	1.94–6.70
Combined T stages						
T_1_ + T_2_	1			1		
T_3_ + T_4_	0.001^*^	5.87	2.13–16.21	0.004^*^	8.25	2.00–34.12
Combined N stages						
N_0_	1			1		
N_1_ + N_2_	< 0.001^*^	3.74	2.19–6.38	< 0.001^*^	3.48	1.86–6.52
TNM stages						
I + II	1			1		
III	< 0.001^*^	3.78	2.23–6.42	< 0.001^*^	3.44	1.85–6.39
PDS						
0	1			1		
1	0.005^*^	0.43	0.24–0.77	0.003^*^	0.36	0.18–0.70
2	< 0.001^*^	0.28	0.14–0.54	< 0.001^*^	0.12	0.05–0.32

*With significant statistical difference

**Table 3:** Multivariate tests for risk factors for DFS and OS

	DFS			OS		
	P	HR	95%CI	P	HR	95%CI
Tumor deposits						
Without	1					
With	0.002^*^	2.85	1.48–5.51			
CEA level						
Normal	1			1		
Elevated	0.004^*^	2.20	1.29–3.74	0.012^*^	2.29	1.20–4.37
TNM stages						
I + II	1			1		
III	0.001^*^	2.59	1.45–4.65	0.017^*^	2.33	1.16–4.68
PDS						
0	1			1		
1	0.049^*^	0.54	0.30–1.00	0.020^*^	0.44	0.22–0.88
2	0.009^*^	0.40	0.20–0.79	< 0.001^*^	0.17	0.06–0.45

*With significant statistical difference

## Discussion

In this study, we found that the PDS was a useful prognostic marker for CRC patients after curative surgery. PDS 0 patients had the worst outcome, and OS could be well distinguished among PDS subgroups. To the best of our knowledge, this is the first report about the prognostic value of PDS in cancer.

The ultimate survival of cancer patients is determined by many factors in addition to the presence of cancer cells. PNI is a marker that combines nutritional status and anticancer immunity in patients, and it is plausible that its prognostic efficacy would be superior to that of other markers that only consider a single factor. In fact, some previous studies have validated this hypothesis. For example, Imai et al. assessed 717 consecutive hepatocellular cancer patients after curative resection and found that PNI was superior to controlling the nutritional status score, NLR, PLR and Glasgow prognostic score in both DFS and OS; PNI was the only independent risk factor in multivariate analyses [14]. Consistent with this finding, Komura et al. enrolled 308 epithelial ovarian cancer patients and found that pretreatment PNI was better than single platelet count in predicting DFS [15]. As noted for CRC, numerous studies have investigated the role of PNI and other systematic inflammatory prognostic indicators. For example, Sato et al. collected 72 stage II-III obstructive patients and found that PNI was the only independent risk factor for DFS and OS compared to NLR, LMR and PLR [38]; accordingly, Maruyama et al. included 197 stage IIA patients and found that PNI was the only independent risk factor in contrast to NLR and PLR [16]. Nonetheless, PNI alone is still limited by its relatively low prognostic efficacy, as mentioned previously [17–19], and could be further improved when some factors that reflect cancer cell features are taken into consideration based on our hypothesis. With the exception of direct quantification of the CTCs, the tumor markers were a good label of malignant cells. CEA is a classical tumor marker that is mainly released by colorectal cancer cells [39, 40] and is a good indicator of the aggressiveness of these cells [41, 42]. Previously, some investigators have tried to combine PNI with CEA to improve the prognostic efficacy. For example, Uejima et al. included 135 stage II patients and found that a combination of PNI and CEA exhibited good prognostic efficacy, but the 5-year DFS rates in the CEA^low^/PNI^high^, CEA^high^/PNI^high^ and CEA^low^/PNI^low^ groups were 100%, 100% and 97.4%, respectively, making it difficult to distinguish the survival differences [43]. Similarly, Xu et al. enrolled 513 stage II–III patients and reported that PNI and CEA represent a superior combination, but the 1- and 3-year OS rates in the aforementioned patient subgroups were undistinguishable [44]. Taking into account these results, it was suggested that an additional indicator that was more reliable than CEA was needed. DD has been consistently reported as a tumor marker in CRC [28] and is also a robust prognostic predictor in CRC [29, 30]. Interestingly, the AUC of DD was higher than that of CEA (0.85 vs. 0.72) with a significantly better sensitivity (88.0% vs. 65.2%) in CRC patients after curative resection [45]. Although no related reports have described the use of DD in combination with other prognostic makers in CRC, one study indicated that a combination of DD with NLR could be a useful prognostic indicator in non-small cell lung cancer (NSCLC), and the 5-year OS rates were distinguishable among the subgroups (23.5% *vs.* 34.2% *vs.* 50.0%) [46]. In our study, the 3-year OS rates in the PDS 0, 1 and 2 subgroups were 95.35%, 75.51% and 59.62%, respectively (P < 0.001), and could be effectively separated. However, when patients were divided into 4 subgroups (PNI^high^/DD^low^, PNI^high^/DD^high^, PNI^low^/DD^low^ and PNI^low^/DD^high^) as noted in previous studies (only those stage II–III) [43, 44], the 3-year OS rates were 95.35%, 80.95%, 71.43%, and 58.82%, respectively (P = 0.001, data not shown), and it was also difficult to separate the survival differences among the subgroups except for the comparisons between the PNI^high^/DD^low^ and PNI^low^/DD^high^ as well as the PNI^high^/DD^high^ and PNI^low^/DD^high^ subgroups. These results were partly consistent with previous studies [43, 44].

Interestingly, it was proposed that cancer is actually a stem cell disease [47]. Colorectal cancer stem cells (CCSCs) identified by specific surface markers, such as CD44 and CD133, have been extensively studied [48]. In a previous study, CCSCs were thought to be the ultimate reason for cancer initiation, dissemination and recurrence, and eradiation of these cells represents a key approach to cure the disease [48, 49]. Of note, cancer dissemination could occur at the very beginning in CRC [50], and these cells in the circulating system (CTCs) exhibit some features of CCSCs [51]. In addition, cancer-promoted inflammation also plays a key role in determining CRC development [52], and inflammatory cytokines, such as interleukin-6 (IL-6) and IL-1, are significantly elevated in CRC patients [53–55], which could have an important role in supporting CCSCs [56, 57]. Interestingly, albumin synthesis could be significantly decreased under inflammatory conditions, and IL-1 was postulated to be an important mediator [58]. Additionally, IL-6 contributes to the decreased expression of albumin genes through the activation of tyrosine kinase [59]. Lymphocytes are the major players in adaptive anticancer immunity [60] and specifically recognize and eradicate CCSCs [61]. Although not reported in CRC, it was found that the proportion of circulating lymphocytes could be decreased under inflammatory conditions [62, 63] in addition to the abnormal immune-suppression function induced by these cytokines [64]. In recent years, accumulating evidence has indicated that CTCs are the key source of relapse in CRC [65, 66]. Interestingly, DD is correlated with CTCs and is an essential accompaniment of these cells [67]. More importantly, the level of DD correlated with CTCs in breast cancer and non-small cell lung cancer [33, 34]. Based on these facts, it would be plausible that patients with PDS 0 have relatively low PNI and high DD levels that are equal to strong cancer-promoting inflammation and sustained high counts of CTCs, which would correlate with poor outcome. However, studies that directly and concurrently analyze the levels of inflammatory cytokines and PNI and DD remain lacking.

Clinically, adjuvant chemotherapy was conventionally recommended for high–risk stage II and stage III CRC patients [68, 69], which could greatly improve the DFS and OS [68–71]. Previously, pT4, lymph-vascular or perineural invasion, perforation or obstruction presentation, poorly differentiated histology, or lymph node harvest less than 12 were well acknowledged as risk factors in CRC [72]. In our study, PDS was found to be useful in prognostic prediction and PDS 0 patients displayed significant poor DFS and OS compared to other subgroups, we speculate maybe it could be considered as an additional risk factor for decision-making approach for the patients in practice; however, randomized controlled trails are necessary to confirm our speculation. In addition, taking into consideration that PDS was an easily accessible indicator, it may also valuable in monitoring the treatment response of metastatic disease to systemic therapies like CEA [73]; however, more studies are needed to validate its role in such a scenario.

There are some limitations to our study. First, it was a retrospective study with a relatively small sample size, and potential biases cannot be excluded. Second, information on adjuvant chemotherapy was insufficient, and it was notable that both the albumin levels and absolute lymphocyte counts could be altered by these therapies [74, 75]; thus, long-term PDS measurements and assessment of its prognostic value in these patients should be performed in the future.

## Conclusion

Overall, our study suggested that PDS was a useful prognostic indicator in CRC patients after curative surgery and that PDS 0 patients exhibit inferior survival. Additional studies are needed to validate these findings in the future.

## Data Availability

The datasets generated or analyzed during the current study are available from the corresponding author (BY) on reasonable request.

## Abbreviations

PNI: Prognostic nutritional index
DD: D-dimer
CRC: Colorectal cancer
PDS: PNI and DD score
DFS: Disease free survival
OS: Overall survival
AUC: Area under the curve
CTCs: Circulating tumor cells
CEA: Carcinoembryonic antigen
NLR: Neutrophil to lymphocyte ratio
LMR: Lymphocyte to monocyte ratio
PLR: Platelet to lymphocyte ratio
CCSCs: Colorectal cancer stem cells
IL-6: Interleukin-6

## References

[1] Xia C, Dong X, Li H, Cao M, Sun D, He S, Yang F, Yan X, Zhang S, Li N (2022). Cancer statistics in China and United States, 2022: profiles, trends, and determinants. Chin Med J (Engl).

[2] GBD 2019 Colorectal Cancer Collaborators. Global, regional, and national burden of colorectal cancer and its risk factors, 1990–2019: a systematic analysis for the Global Burden of Disease Study 2019. Lancet Gastroenterol Hepatol. 2022;7(7):627–47. 10.1016/S2468-1253(22)00044-9 PMC9192760 35397795

[3] Cardoso R, Guo F, Heisser T, Hackl M, Ihle P, De Schutter H, Van Damme N, Valerianova Z, Atanasov T, Májek O (2021). Colorectal cancer incidence, mortality, and stage distribution in European countries in the colorectal cancer screening era: an international population-based study. Lancet Oncol.

[4] Meyerhardt JA, Mayer RJ (2005). Systemic therapy for colorectal cancer. N Engl J Med.

[5] Zitvogel L, Pietrocola F, Kroemer G (2017). Nutrition, inflammation and cancer. Nat Immunol.

[6] Grivennikov SI, Greten FR, Karin M (2010). Immunity, inflammation, and cancer. Cell.

[7] Bahçeci A, Kötek Sedef A, Işik D (2022). The prognostic values of prognostic nutritional index in extensive-stage small-cell lung cancer. Anticancer Drugs.

[8] Tang M, Jia Z, Zhang J (2021). The prognostic role of prognostic nutritional index in nasopharyngeal carcinoma: A systematic review and meta-analysis. Int J Clin Oncol.

[9] Man Z, Pang Q, Zhou L, Wang Y, Hu X, Yang S, Jin H, Liu H (2018). Prognostic significance of preoperative prognostic nutritional index in hepatocellular carcinoma: a meta-analysis. HPB.

[10] Mohri T, Mohri Y, Shigemori T, Takeuchi K, Itoh Y, Kato T (2016). Impact of prognostic nutritional index on long-term outcomes in patients with breast cancer. World J Surg Oncol.

[11] Okubo K, Arigami T, Matsushita D, Tanaka T, Tsuruda Y, Noda M, Sasaki K, Mori S, Kurahara H, Ohtsuka T (2021). Clinical impact of the prognostic nutritional index as a predictor of outcomes in patients with stage II/III gastric cancer: a retrospective cohort study. Oncology.

[12] Xie H, Wei L, Yuan G, Liu M, Tang S, Gan J (2022). Prognostic value of prognostic nutritional index in patients with colorectal cancer undergoing surgical treatment. Front Nutr.

[13] Senger AS, Dincer M, Uzun O, Gulmez S, Avan D, Ofluoglu CB, Polat E, Duman M (2022). Impact of preoperative prognostic nutritional index levels on morbidity in colorectal cancer surgery. Ann Ital Chir.

[14] Imai D, Maeda T, Shimokawa M, Wang H, Yoshiya S, Takeishi K, Itoh S, Harada N, Ikegami T, Yoshizumi T (2020). Prognostic nutritional index is superior as a predictor of prognosis among various inflammation-based prognostic scores in patients with hepatocellular carcinoma after curative resection. Hepatol Res.

[15] Komura N, Mabuchi S, Yokoi E, Shimura K, Kawano M, Matsumoto Y, Kimura T (2019). Pre-treatment prognostic nutritional index is superior to platelet count in predicting disease-specific survival in patients with epithelial ovarian cancer. Int J Gynecol Cancer.

[16] Maruyama T, Shimoda M, Hakoda H, Sako A, Ueda K, Suzuki S (2021). Preoperative prognostic nutritional index predicts risk of recurrence after curative resection for stage IIA colon cancer. Am J Surg.

[17] Peng J, Zhang R, Zhao Y, Wu X, Chen G, Wan D, Lu Z, Pan Z (2017). Prognostic value of preoperative prognostic nutritional index and its associations with systemic inflammatory response markers in patients with stage III colon cancer. Chin J Cancer.

[18] Tominaga T, Nagasaki T, Akiyoshi T, Fukunaga Y, Honma S, Nagaoka T, Matsui S, Minami H, Miyanari S, Yamaguchi T (2020). Prognostic nutritional index and postoperative outcomes in patients with colon cancer after laparoscopic surgery. Surg Today.

[19] Ucar G, Ergun Y, Acikgoz Y, Uncu D (2020). The prognostic value of the prognostic nutritional index in patients with metastatic colorectal cancer. Asia Pac J Clin Oncol.

[20] Wang Y, Zhu Z, Li C, Ma Y, You Q, Li Z, Zhang H, Song H, Xue Y (2019). Prognostic significance of preoperative albumin-to-globulin ratio and prognostic nutritional index combined score in Siewert type 3 adenocarcinoma of esophagogastric junction. Cancer Manag Res.

[21] Wang B, Jiang XW, Tian DL, Zhou N, Geng W (2020). Combination of haemoglobin and prognostic nutritional index predicts the prognosis of postoperative radiotherapy for esophageal squamous cell carcinoma. Cancer Manag Res.

[22] Uen YH, Lu CY, Tsai HL, Yu FJ, Huang MY, Cheng TL, Lin SR, Wang JY (2008). Persistent presence of postoperative circulating tumor cells is a poor prognostic factor for patients with stage I-III colorectal cancer after curative resection. Ann Surg Oncol.

[23] Lu CY, Uen YH, Tsai HL, Chuang SC, Hou MF, Wu DC, Juo SH, Lin SR, Wang JY (2011). Molecular detection of persistent postoperative circulating tumour cells in stages II and III colon cancer patients via multiple blood sampling: prognostic significance of detection for early relapse. Br J Cancer.

[24] Yang C, Wei C, Wang S, Han S, Shi D, Zhang C, Lin X, Dou R, Xiong B (2019). Combined features based on preoperative controlling nutritional status score and circulating tumour cell status predict prognosis for colorectal cancer patients treated with curative resection. Int J Biol Sci.

[25] Ma M, Cao R, Wang W, Wang B, Yang Y, Huang Y, Zhao G, Ye L (2021). The D-dimer level predicts the prognosis in patients with lung cancer: a systematic review and meta-analysis. J Cardiothorac Surg.

[26] Kim EY, Song KY (2021). Prognostic value of D-dimer levels in patients with gastric cancer undergoing gastrectomy. Surg Oncol.

[27] Batschauer APB, Figueiredo CP, Bueno EC, Ribeiro MA, Dusse LMS, Fernandes AP, Gomes KB, Carvalho MG (2010). D-dimer as a possible prognostic marker of operable hormone receptor-negative breast cancer. Ann Oncol.

[28] Oya M, Akiyama Y, Yanagida T, Akao S, Ishikawa H (1998). Plasma D-dimer level in patients with colorectal cancer: its role as a tumor marker. Surg Today.

[29] Oya M, Akiyama Y, Okuyama T, Ishikawa H (2001). High preoperative plasma D-dimer level is associated with advanced tumor stage and short survival after curative resection in patients with colorectal cancer. Jpn J Clin Oncol.

[30] Kilic M, Yoldas O, Keskek M, Ertan T, Tez M, Gocmen E, Koc M (2008). Prognostic value of plasma D-dimer levels in patients with colorectal cancer. Colorectal Dis.

[31] Lu SL, Ye ZH, Ling T, Liang SY, Li H, Tang XZ, Xu YS, Tang WZ (2017). High pretreatment plasma D-dimer predicts poor survival of colorectal cancer: insight from a meta-analysis of observational studies. Oncotarget.

[32] Blackwell K, Hurwitz H, Liebérman G, Novotny W, Snyder S, Dewhirst M, Greenberg C (2004). Circulating D-dimer levels are better predictors of overall survival and disease progression than carcinoembryonic antigen levels in patients with metastatic colorectal carcinoma. Cancer.

[33] Mego M, Zuo Z, Gao H, Cohen EN, Giordano A, Tin S, Anfossi S, Jackson S, Woodward W, Ueno NT (2015). Circulating tumour cells are linked to plasma D-dimer levels in patients with metastatic breast cancer. Thromb Haemost.

[34] Wang J, Liu H, Sun X, Liu Q, Han S, Zhu L (2021). Correlation between circulating tumor cells and plasma D-dimer and clinicopathological characteristics of patients with non-small cell lung cancer. J Coll Physicians Surg Pak.

[35] Huang X, Huan Y, Liu L, Ye Q, Guo J, Yan B (2022). Preoperative low absolute lymphocyte count to fibrinogen ratio correlated with poor survival in nonmetastatic colorectal cancer. World J Surg Oncol.

[36] Zhang Y, Liu Y, Qiu X, Yan B (2021). Concurrent comparison of the prognostic values of tumor budding, tumor stroma ratio, tumor infiltrating pattern and lymphocyte-to-monocyte ratio in colorectal cancer patients. Technol Cancer Res Treat.

[37] Hailun X, Huang S, Yuan G, Tang S, Gan J (2021). Prognostic significance of preoperative fibrinogen-to-prealbumin ratio in patients with stage I-III colorectal cancer undergoing surgical resection: a retrospective cohort study. BioMed Res Int.

[38] Sato R, Oikawa M, Kakita T, Okada T, Abe T, Yazawa T, Tsuchiya H, Akazawa N, Sato M, Ohira T (2020). The prognostic value of the prognostic nutritional index and inflammation-based markers in obstructive colorectal cancer. Surg Today.

[39] Gold P, Freedman SO (1965). Demonstration of tumor-specific antigens in huamn colonic carcinoma by immunological tolerance and absorption techniques. J Exp Med.

[40] Goldstein MJ, Mitchell EP (2005). Carcinoembryonic antigen in the staging and follow-up of patients with colorectal cancer. Cancer Invest.

[41] Bajenova O, Gorbunova A, Evsyukov I, Rayko M, Gapon S, Bozhokina E, Shishkin A, O'Brien SJ (2016). The genome-wide analysis of carcinoembryonic antigen signaling by colorectal cancer cells using RNA sequencing. PLoS ONE.

[42] Huang EY, Chang JC, Chen HH, Hsu CY, Hsu HC, Wu KL (2018). Carcinoembryonic antigen as a marker of radioresistance in colorectal cancer: a potential role of macrophages. BMC Cancer.

[43] Uejima C, Saito H, Tada Y, Tanio A, Murakami Y, Yamamoto M, Matsunaga T, Fukumoto Y, Tokuyasu N, Takano S (2021). The Combination of prognostic nutritional indicator and serum carcinoembryonic antigen is useful in predicting postoperative recurrence in stage II colorectal cancer. Yonago Acta Med.

[44] Xu YS, Liu G, Zhao C, Lu SL, Long CY, Zhong HG, Chen Y, Huang LX, Liang Z (2021). Prognostic value of combined preoperative carcinoembryonic antigen and prognostic nutritional index in patients with stage II-III colon cancer. Front Surg.

[45] Guo Y, Chen F, Cui W (2018). Usefulness of plasma D-dimer level for monitoring development of distant organ metastasis in colorectal cancer patients after curative resection. Cancer Manag Res.

[46] Liang HG, Gao K, Jia R, Li J, Wang C (2019). Prognostic significance of the combination of preoperative fibrinogen and the neutrophil-lymphocyte ratio in patients with non-small cell lung cancer following surgical resection. Oncol Lett.

[47] Alison MR, Poulsom R, Forbes S, Wright NA (2002). An introduction to stem cells. J Pathol.

[48] Hervieu C, Christou N, Battu S, Mathonnet M (2021). The role of cancer stem cells in colorectal cancer: from the basics to novel clinical trials. Cancers (Basel).

[49] Gupta R, Bhatt LK, Johnston TP, Prabhavalkar KS (2019). Colon cancer stem cells: Potential target for the treatment of colorectal cancer. Cancer Biol Ther.

[50] Hu Z, Ding J, Ma Z, Sun R, Seoane JA, Scott Shaffer J, Suarez CJ, Berghoff AS, Cremolini C, Falcone A (2019). Quantitative evidence for early metastatic seeding in colorectal cancer. Nat Genet.

[51] Schölch S, García SA, Iwata N, Niemietz T, Betzler AM, Nanduri LK, Bork U, Kahlert C, Thepkaysone M-L, Swiersy A (2016). Circulating tumor cells exhibit stem cell characteristics in an orthotopic mouse model of colorectal cancer. Oncotarget.

[52] Hanahan D, Weinberg RA (2011). Hallmarks of cancer: the next generation. Cell.

[53] Belluco C, Nitti D, Frantz M, Toppan P, Basso D, Plebani M, Lise M, Jessup JM (2000). Interleukin-6 blood level is associated with circulating carcinoembryonic antigen and prognosis in patients with colorectal cancer. Ann Surg Oncol.

[54] Guthrie GJ, Roxburgh CS, Richards CH, Horgan PG, McMillan DC (2013). Circulating IL-6 concentrations link tumour necrosis and systemic and local inflammatory responses in patients undergoing resection for colorectal cancer. Br J Cancer.

[55] Jocić M, Arsenijević N, Gajović N, Jurišević M, Jovanović I, Jovanović M, Zdravković N, Marić V, Jovanović M (2022). Anemia of inflammation in patients with colorectal cancer: Correlation with interleukin-1, interleukin-33 and galectin-1. J Med Biochem.

[56] Huynh PT, Beswick EJ, Coronado YA, Johnson P, O'Connell MR, Watts T, Singh P, Qiu S, Morris K, Powell DW (2016). CD90(+) stromal cells are the major source of IL-6, which supports cancer stem-like cells and inflammation in colorectal cancer. Int J Cancer.

[57] Kim B, Seo Y, Kwon JH, Shin Y, Kim S, Park SJ, Park JJ, Cheon JH, Kim WH, Il KT (2021). IL-6 and IL-8, secreted by myofibroblasts in the tumor microenvironment, activate HES1 to expand the cancer stem cell population in early colorectal tumor. Mol Carcinog.

[58] Moshage HJ, Janssen JA, Franssen JH, Hafkenscheid JC, Yap SH (1987). Study of the molecular mechanism of decreased liver synthesis of albumin in inflammation. J Clin Invest.

[59] Huang Y, Shinzawa H, Togashi H, Takahashi T, Kuzumaki T, Otsu K, Ishikawa K (1995). Interleukin-6 down-regulates expressions of the aldolase B and albumin genes through a pathway involving the activation of tyrosine kinase. Arch Biochem Biophys.

[60] Disis ML (2010). Immune regulation of cancer. J Clin Oncol.

[61] Inoda S, Hirohashi Y, Torigoe T, Morita R, Takahashi A, Asanuma H, Nakatsugawa M, Nishizawa S, Tamura Y, Tsuruma T (2011). Cytotoxic T lymphocytes efficiently recognize human colon cancer stem-like cells. Am J Pathol.

[62] Jewell AP, Worman CP, Giles FJ, Goldstone AH (1997). Serum levels of TNF, IL-6 and sCD23 correlate with changes in lymphocyte count in patients with B-cell chronic lymphocytic leukaemia receiving interferon-alpha therapy. Leuk Lymphoma.

[63] Yu Y, Wang S, Su N, Pan S, Tu B, Zhao J, Shen Y, Qiu Q, Liu X, Luan J (2022). Increased circulating levels of CRP and IL-6 and decreased frequencies of T and B lymphocyte subsets are associated with immune-related adverse events during combination therapy with PD-1 inhibitors for liver cancer. Front Oncol.

[64] Bent EH, Millán-Barea LR, Zhuang I, Goulet DR, Fröse J, Hemann MT (2021). Microenvironmental IL-6 inhibits anti-cancer immune responses generated by cytotoxic chemotherapy. Nat Commun.

[65] Zahran AM, Rayan A, Fakhry H, Attia AM, Ashmawy AM, Soliman A, Elkady A, Hetta HF (2019). Pretreatment detection of circulating and tissue CD133(+) CD44(+) cancer stem cells as a prognostic factor affecting the outcomes in Egyptian patients with colorectal cancer. Cancer Manag Res.

[66] Grillet F, Bayet E, Villeronce O, Zappia L, Lagerqvist EL, Lunke S, Charafe-Jauffret E, Pham K, Molck C, Rolland N (2017). Circulating tumour cells from patients with colorectal cancer have cancer stem cell hallmarks in *ex vivo* culture. Gut.

[67] Diao D, Cheng Y, Song Y, Zhang H, Zhou Z, Dang C (2017). D-dimer is an essential accompaniment of circulating tumor cells in gastric cancer. BMC Cancer.

[68] Souglakos J, Boukovinas I, Kakolyris S, Xynogalos S, Ziras N, Athanasiadis A (2019). Three- versus six-month adjuvant FOLFOX or CAPOX for high-risk stage II and stage III colon cancer patients: the efficacy results of Hellenic Oncology Research Group (HORG) participation to the International Duration Evaluation of Adjuvant Chemotherapy (IDEA) project. Ann Oncol.

[69] Kim ST, Kim SY, Lee J, Yun SH, Kim HC, Lee WY (2022). Oxaliplatin (3 months v 6 months) With 6 Months of Fluoropyrimidine as Adjuvant Therapy in Patients With Stage II/III Colon Cancer: KCSG CO09-07. J Clin Oncol.

[70] André T, Boni C, Navarro M, Tabernero J, Hickish T, Topham C (2009). Improved overall survival with oxaliplatin, fluorouracil, and leucovorin as adjuvant treatment in stage II or III colon cancer in the MOSAIC trial. J Clin Oncol.

[71] Grant RRC, Khan TM, Gregory SN, Coakley BA, Hernandez JM, Davis JL, Blakely AM (2022). Adjuvant chemotherapy is associated with improved overall survival in select patients with Stage II colon cancer: A National Cancer Database analysis. J Surg Oncol.

[72] Dienstmann R, Salazar R, Tabernero J (2015). Personalizing colon cancer adjuvant therapy: selecting optimal treatments for individual patients. J Clin Oncol.

[73] Locker GY, Hamilton S, Harris J, Jessup JM, Kemeny N, Macdonald JS, Somerfield MR, Hayes DF, Bast RC. ASCO 2006 update of recommendations for the use of tumor markers in gastrointestinal cancer. J Clin Oncol. 2006;24(33):5313–27. 10.1200/JCO.2006.08.2644 17060676

[74] Wang X, Han H, Duan Q, Khan U, Hu Y, Yao X (2014). Changes of serum albumin level and systemic inflammatory response in inoperable non-small cell lung cancer patients after chemotherapy. J Cancer Res Ther.

[75] Wang W, Wang Y, Cao Z (2021). Changes of proportions of circulating lymphocyte subsets in cancer patients after chemotherapy. Transl Cancer Res.

